# The association between daily concentrations of air pollution and visits to a psychiatric emergency unit: a case-crossover study

**DOI:** 10.1186/s12940-017-0348-8

**Published:** 2018-01-10

**Authors:** Anna Oudin, Daniel Oudin Åström, Peter Asplund, Steinn Steingrimsson, Zoltan Szabo, Hanne Krage Carlsen

**Affiliations:** 1 Occupational and Environmental Medicine, Lund University, Medicon Village, Byggnad 402A, Scheelevägen 2, 223 63 Lund, Sweden; 2 Occupational and Environmental Medicine, Umeå University, Umeå, Sweden; 3 Center for Primary Health Care Research, Department of Clinical Sciences, Lund University, Malmö, Sweden; 4 Psykiatri Affektiva, Sahlgrenska University Hospital, Gothenburg, Sweden; 5 CELAM - Centre for Ethics, Law and Mental Health, Sahlgrenska University Hospital, Gothenburg, Sweden; 6 Environment and Natural Resources, University of Iceland, Reykjavík, Iceland; 7 Occupational and Environmental Medicine, Gothenburg University, Gothenburg, Sweden

**Keywords:** Air pollution, Particles, Psychiatric disorders, Mental distress, Environmental epidemiology, Acute effects of air pollution

## Abstract

**Background:**

Air pollution is one of the leading causes of mortality and morbidity worldwide. Experimental studies, and a few epidemiological studies, suggest that air pollution may cause acute exacerbation of psychiatric disorders, and even increase the rate of suicide attempts, but epidemiological studies on air pollution in association with psychiatric disorders are still few. Our aim was to investigate associations between daily fluctuations in air pollution concentrations and the daily number of visits to a psychiatric emergency unit.

**Methods:**

Data from Sahlgrenska University Hospital, Gothenburg, Sweden, on the daily number of visits to the Psychiatric emergency unit were combined with daily data on monitored concentrations of respirable particulate matter(PM_10_), ozone(O_3_), nitrogen dioxides(NO_2_) and temperature between 1st July 2012 and 31st December 2016. We used a case-crossover design to analyze data with conditional Poisson regression models allowing for over-dispersion. We stratified data on season.

**Results:**

Visits increased with increasing PM_10_ levels during the warmer season (April to September) in both single-pollutant and two-pollutant models. For example, an increase of 3.6% (95% Confidence Interval, CI, 0.4–7.0%) was observed with a 10 μg/m3 increase in PM_10_ adjusted for NO_2_. In the three-pollutant models (adjusting for NO_2_ and O_3_ simultaneously) the increase was 3.3% (95% CI, −0.2-6.9). There were no clear associations between the outcome and NO_2_, O_3_, or PM_10_ during the colder season (October to March).

**Conclusions:**

Ambient air particle concentrations were associated with the number of visits to the Psychiatric emergency unit in the warm season. The results were only borderline statistically significant in the fully adjusted (three-pollutant) models in this small study. The observation could be interpreted as indicative of air pollution as either exacerbating an underlying psychiatric disorder, or increasing mental distress, even in areas with comparatively low levels of air pollution. In combination with the severe impact of psychiatric disorders and mental distress on society and individuals, our results are a strong warrant for future research in this area.

**Electronic supplementary material:**

The online version of this article (10.1186/s12940-017-0348-8) contains supplementary material, which is available to authorized users.

## Background

Psychiatric disorders and mental distress are major public health problems, and may severely influence individuals’ potential to live fulfilling and productive lives. A common psychiatric disorder such as unipolar depression causes one of the greatest burden of disease worldwide [1], and in Sweden psychiatric disorders are estimated to cost society 70 billion SEK annually or 2.8% of the Gross Domestic Product [2] by the Organization for Economic Co-operation and Development (OECD). Given the substantial personal and societal burden from psychiatric disorders, it is imperative to identify modifiable factors. One important environmental exposure, for which there is emerging evidence of a relation to psychiatric disorders or mental distress, is ambient air pollution.

Ambient air pollution is a complex mixture of unwanted airborne compounds produced mainly by human activity, e.g. traffic, and is one of the biggest health threats of our time [3]. Oberdörster and Utell first suggested that the central nervous system (CNS) might be vulnerable to ultrafine ambient particulate matter through the respiratory tract either via the olfactory bulb or alveolar-capillary transmission of the substance into the blood stream after which it may cross the blood-brain barrier [4]. Even below current air quality guideline levels air pollution exposure is now an established risk factor for stroke [5]. Day-to day variation in air pollution levels is a risk factor for recurrent stroke [6, 7]. Chronic exposure to air pollution is a risk factor for incident stroke [8] and an emerging risk factor for dementia and cognitive decline [7, 9–11]. We recently observed associations between neighborhood annual concentrations of air pollution and dispensed psychotropic medications in children in Swedish children and adolescents [12]. Associations between long-term exposure to air pollution and perceived stress and prevalent anxiety, [13, 14] with risk of schizophrenia, [15] and with depressive and anxiety symptoms [16] have also been reported. In addition to effects of long-term exposure, acute associations between ambient air pollution and psychiatric emergency room visits have been reported [17–19]. Acute associations between air pollution and suicide attempts, [20] air pollution and suicide completion, [21, 22] air pollution and daily hospital admissions for psychiatric disorders [23], psychosis morbidity, [24] and air pollution and aggravated depressive symptoms [25–27] have also been reported.

Even though the number of epidemiological studies are quite few, experimental studies show that an association between air pollution exposure and worsening in psychiatric disorders or mental distress is plausible [28]. Suggested pathways include particles entering the CNS and depositing there, which may lead to inflammation or increased systemic inflammation which may affect CNS functions [29, 30]. Exposure to diesel particles have been shown to activate microglia, which can produce neurotoxicity via oxidative stress [29]. Both oxidative stress [31, 32] and systemic inflammation [33] may induce anxiety-like behaviors in mice and rats. Fonken and colleagues exposed mice to PM_2.5_ and observed that apical dendritic spine density and dendritic branching were decreased in the hippocampal CA1 and CA3 regions in exposed mice [34]. This suggests that exposure to particulate air pollution can alter brain regions involved in affective responses at levels typically present in many cities worldwide. Thomson and colleagues exposed rats to particulate matter and ozone, and saw that pollutant exposure increased the glucocorticoid corticosterone and plasma levels of adrenocorticotropic hormone meaning that the hypothalamic-pituitary-adrenal axis was activated [35]. These recent studies suggest that air pollution exposure had acute effects while both chronic activation and inappropriate regulation of the hypothalamic-pituitary-adrenal axis and inflammation were associated with worsening in depressive symptoms [36].

Considering the severe impact of psychiatric disorders and mental distress on society, a possible association with air pollution, a preventable exposure, deserves special attention. In the present study, we aim to investigate acute effects of air pollution on worsening of psychiatric disorders or of mental distress, using daily levels of air pollution concentrations together with the daily number of Gothenburg municipality residents who seek care at the psychiatric emergency department at Sahlgrenska University Hospital in Gothenburg.

## Methods

### Data

#### Psychiatric emergency visit (PEV) data

The psychiatric emergency department visit data were provided by Sahlgrenska University Hospital for the time period 1st of July 2012 until 24th of November 2016. The psychiatric emergency department at Sahlgrenska University Hospital, is an always-open walk-in clinic located at Östra Sjukhuset. Here, individuals with acute exacerbation of an underlying psychiatric disorder, or first contact due to psychiatric disorder, or individuals with severe psychosocial stress (mental distress) not due to a psychiatric disorder can seek health care. Although many visitors have been referred from other health care providers e.g. primary health care a referral is not needed due to an open-door policy. There is a nominal fee for seeking care. Some individuals may visit due to non-emergency causes e.g. prescription renewal albeit this type of visits constitutes a minority. Daily counts of digitally registered visits to the psychiatric emergency department (PEVs in the following) were collected and we included visits for those residing in the municipality/region of Gothenburg in the current analysis. We did not have data on age, sex or diagnosis or cause for visit for this study, only the number of PEVs per day.

#### Air pollution data

Daily levels of respirable particulate matter (PM_10_), ozone (O_3_), and nitrogen dioxides (NO_2_) for the study period were obtained from the Swedish Environmental Institute and Gothenburg Municipality Environmental Department from the measuring station at Femman house – a centrally located urban background station. Data on PM_2.5_ was available but as large proportion of observations were missing we decided against using this data. We scaled exposure so that all results presented are associated with a 10 μg/m^3^ air pollutant concentrations increase. In extra analyses, we also analyzed intra-quartile-increases (IQRs).

#### Temperature data

The Swedish Meteorological and Hydrological Institute, provided daily mean temperatures obtained at the meteorological station Göteborg A (located near the air pollution measuring station) (Latitude/Longitude 57.7157;11.9925) (http://opendata-download-metobs.smhi.se/explore/?parameter=0. Accessed June 26, 2017). The meteorological data was complete during the study period.

#### Statistical methods

To investigate the short term effect of air pollutants on psychiatric emergency visits (PEVs) we used a case-crossover design, where each individual serves as their own control. The case-crossover design thus adjusts for individual time-invariant confounders [37]. We used three to four control days per PEV, selected within the month, and year, of the date of PEV, and matched on day of week. This selection of control days controls for both seasonality and trends over time as well as a potential effect of day-of-week on PEV counts. In order to avoid bias due to overlapping time-periods we used fixed and disjoint reference periods (calendar months) [38]. A Poisson regression model with a stratum variable yields identical results to those generated from a conditional logistic regression when there is a common exposure across individuals, as is the case in our study where all individuals are assumed to have the same air pollution exposure [39, 40]. We ran conditional Poisson regression models allowing for over-dispersion. Initially, basic models, including only the lag0 of the air pollutants were used. We then adjusted the basic model for temperature (daily mean temperature as well as daily mean temperature fitted as a natural cubic spline with three degrees of freedom) and a binary variable indicating public Swedish holidays (Yes/No). We compared models using Akaike Information Criteria for QuasiPoisson (QAIC). We also ran multi-pollutant models, both crude and adjusted as explained above.

In the main analyses, we used complete-case data, thus excluding all missing observations. In a sensitivity analysis however, we imputed missing data for the PM_10_-variable (the variable with largest proportion of missing observations).

We stratified all analyses on season, where the warm season was defined as April to September, and the cold season as October to March. We investigated delayed effects beyond lag0 to lag 7, and we performed model checks by visual inspection of normally distributed residuals (Additional file 1: Figures S1 and S2).

All analyses were performed with R version 3.4.0.

## Results

**Fig. 1:**
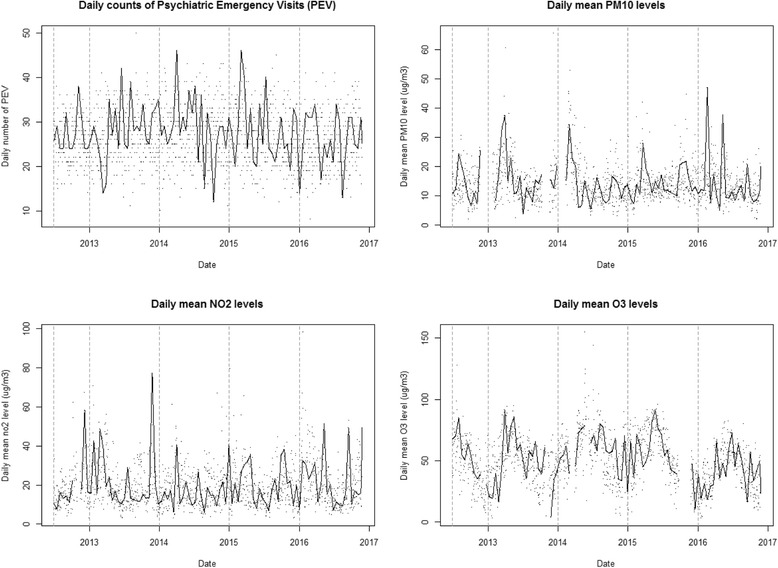
Time series plots for the daily counts of psychiatric emergency visits as well as the mean daily levels of air pollutants (the line is a smooth function so that the patterns are made more visible)

The descriptive statistics for the daily counts of psychiatric emergency visits (PEVs) as well as the air pollutants are presented in Table 1 and Fig. 1. The correlation coefficients between the different air pollutants were generally moderate to low (Additional file 1: Figure S3). The smallest QAIC were provided by the crude model and by the model adjusting for public Swedish holiday and mean temperature as a continuous variable. We thus, for each of the pollutants, present the results from that and as well as the crude model. In single-pollutant unadjusted models the daily lag 0 PM_10_ concentrations were statistically significantly associated with the daily number of PEVs with a 2.1% increase (95% Confidence Interval, CI, of 0.2–4.0%, Table 2). After adjusting for temperature and public holidays, the increase was similar in magnitude at 2.3% (95% CI, 0.4–4.3%, Table 2). In the three pollutant models adjusted also for NO_2_ and O_3_ the increase was 1.4% (95% CI,-0.8–3.7, Table 2). The effect estimates were higher during the warm season (April to September), with an estimated increase of 3.6% (95% CI, 0.4 to 7.0%, Table 3) in the adjusted two-pollutant model, than during the cold season, where the corresponding estimate was 0.5% (95% CI, −2.5-3.5%, Table 4). In the three-pollutant model, the warm season estimate was similar to the two-pollutant model estimates, 3.3% (95% CI, −0.2 to 6.9%, Table 3), but not statistical significant.

**Table 1:** Descriptive statistics for the daily counts of psychiatric emergency visits (PEVs) and air pollutant concentrations (μg/m^3^)

	Mean	SD^a^	Min	Max	Median	Q1^b^	Q3^c^	Missing days
PEV	27	6	8	50	27	22	31	0 (0%)
PM_10_	14.2	6.6	1.8	65.6	12.9	10.0	16.7	153 (9.5%)
NO_2_	19.5	11.0	2.9	98.4	17.1	11.8	24.6	32 (2.0%)
O_3_	50.3	20.0	2.8	155.0	50.2	36.5	63.8	112 (7.0%)
Warmer months (April to September)								
PEV	27	6	11	50	27	22	31	0 (0%)
PM_10_	13.5	5.4	2.0	60.5	12.5	10.1	15.8	7 (0.8%)
NO_2_	17.2	8.7	2.9	56.1	15.4	11.0	21.3	5 (0.6%)
O_3_	57.9	18.1	10.6	155.0	57.4	45.	69.7	15 (1.8%)
Colder months (October to March)								
PEV	27	6	8	46	26	22	31	0 (0%)
PM_10_	15.1	7.8	1.8	65.6	13.7	9.8	18.5	146 (18.6%)
NO_2_	22.0	12.6	2.9	98.4	19.4	13.0	27.9	27 (3.4%)
O_3_	41.3	18.2	2.8	91.3	40.7	27.3	54.1	97 (12.4%)

^a^SD Standard deviation, ^b^Q1 1^st^ quartile, ^c^Q3 3^rd^ quartile

**Table 2:** Percent change in the number of Psychiatric Emergency Visits (PEVs) with their 95% Confidence Intervals for an increase of 10 μg/m^3^ in lag 0 in the air pollutant for Single-pollutant, two-pollutant, and Multi-pollutant models

	Model	PM_10_	NO_2_	O_3_
		Change PEVs (%) (95% CI)	Change PEVs (%) (95% CI)	Change PEVs (%) (95% CI)
Single Pollutant	Crude^a^	2.1 (0.2–4.1)	0.3 (−0.9–1.4)	0.2 (−0.6–0.9)
Single Pollutant	Adjusted^b^	2.3 (0.3–4.3)	0.3 (−0.8–1.5)	0.1 (−0.6–0.9)
Two-pollutants				
PM_10_ + NO_2_	Crude	2.0 (0.0–4.0)	0.5 (−0.8–1.7)	
PM_10_ + NO_2_	Adjusted	2.2 (0.2–4.3)	0.5 (−0.8–1.7)	
PM_10_ + O_3_	Crude	1.7 (−0.4–3.9)		0.3 (−0.5–1.1)
PM_10_ + O_3_	Adjusted	1.9 (−0.03–4.1)		0.3 (−0.5–1.2)
NO_2_ + O_3_	Crude		0.8 (−0.7–2.3)	0.4 (−0.5–1.4)
NO_2_ + O_3_	Adjusted		0.8 (−0.7–2.4)	0.4 (−0.5–1.4)
Three-pollutants				
PM_10_ + NO_2_ + O_3_	Crude	1.4 (−0.8–3.7)	1.2 (−0.4–2.9)	0.7 (−0.3–1.8)
PM_10_ + NO_2_ + O_3_	Adjusted	1.6 (−0.1–3.9)	1.2 (−0.5–2.9)	0.7 (−0.4–1.8)

^a^Model with only air pollutants

^b^Adjusted = Model adjusting for daily mean temperature (continuous) and public Swedish holiday (Yes/No)

**Table 3:** Percent change in the number of Psychiatric Emergency Visits (PEVs) with their 95% Confidence Intervals for an increase of 10 μg/m^3^ in lag 0 in the air pollutant for Single and Multi-pollutant models during the warmer season (April to September)

	Model	PM_10_	NO_2_	O_3_
		Change PEVs (%) (95% CI)	Change PEVs (%) (95% CI)	Change PEVs (%) (95% CI)
Single Pollutant	Crude^q^	3.4 (0.3–6.6)	-0.2 (−2.11.7)	0.7 (−0.3–1.7)
Single Pollutant	Adjusted^b^	3.5 (0.4–6.8)	0.0 (0.981–1.019)	0.6 (−0.5–1.6)
Two-pollutants				
PM_10_ + NO_2_	Crude	3.6 (0.3–6.9)	−0.5 (−2.4–1.4)	
PM_10_ + NO_2_	Adjusted	3.6 (0.4–7.0)	−0.4 (−2.3–1.6)	
PM_10_ + O_3_	Crude	3.0 (0.7–5.4)		0.5 (−0.6–1.5)
PM_10_ + O_3_	Adjusted	3.6 (0.4–7.0)		0.3 (−0.5–1.2)
NO_2_ + O_3_	Crude		0.6 (−1.7–2.9)	0.9 (−0.3–2.1)
NO_2_ + O_3_	Adjusted		0.7 (−1.3–2.7)	0.8–0.4-2.0)
Three-pollutants				
PM_10_ + NO_2_ + O_3_	Crude	3.2 (−0.2–6.8)	−0.1 (−2.5–2.3)	0.4 (−0.9–1.7)
PM_10_ + NO_2_ + O_3_	Adjusted	3.3 (−0.2–6.9)	−0.1 (−2–5-2.4)	0.3 (−1.0–1.6)

^a^Model with only air pollutants

^b^Adjusted = Model adjusting for daily mean temperature (continuous) and public Swedish holiday (Yes/No)

**Table 4:** Percent change in the number of Psychiatric Emergency Visits (PEVs) with their 95% Confidence Intervals for an increase in 10 μg/m^3^ in lag 0 of the air pollutant for Single and Multi-pollutant models during the colder season (October to March)

	Model	PM_10_	NO_2_	O_3_
		Change PEVs (%) (95% CI)	Change PEVs (%) (95% CI)	Change PEVs (%) (95% CI)
Single Pollutant	Crude^a^	1.3 (−1.2–3.9)	0.5 (−0.9–2.0)	−0.4 (−1.6–0.7)
Single Pollutant	Adjusted^b^	1.6 (−0.9–4.3)	0.5 (−1.0–2.0)	−0.3 (−1.6–0.9)
Two-pollutants				
PM_10_ + NO_2_	Crude	1.1 (−1.5–3.8)	1.0 (−0.6–2.7)	
PM_10_ + NO_2_	Adjusted	1.5 (−1.2–4.2)	0.8 (−0.9–2.5)	
PM_10_ + O_3_	Crude	0.7 (−2.2–3.7)		0.7 (−1.3–1.4)
PM_10_ + O_3_	Adjusted	1.0 (−0.2–4.1)		0.2 (−1.1–1.6)
NO_2_ + O_3_	Crude		0.5 (−1.5–2.7)	−0.3 (−1.9–1.3)
NO_2_ + O_3_	Adjusted		0.5 (−2.0–3.0)	−0.3 (−1.9–1.4)
Three-pollutants				
PM_10_ + NO_2_ + O_3_	Crude	0.5 (−2.5–3.5)	2.0 (−0.4–4.5)	0.9 (−1.0–2.8)
PM_10_ + NO_2_ + O_3_	Adjusted	0.8 (−2.3–4.0)	2.0 (−0.6–4.6)	1.1 (−0.9–3.1)

^a^Model with only air pollutants

^b^Adjusted = Model adjusting for daily mean temperature (continuous) and public Swedish holiday (Yes/No)

There were no increases in PEVs associated with increases in NO_2_ or O_3_ (Tables 2, 3 and 4), although there was a tendency for NO_2_ to be associated with an increased number of PEVs during the cold season in the adjusted three-pollutant models, with an increase of 2.0% (95% CI: -0.6-4.6). The associations observed for lag 1 to lag 7 were generally lower than for lag 0 and were not statistically significant (data not shown). When imputing missing data on PM_10_ concentrations, the results were very similar (we estimated, using data for the whole year, a 2.0% increase in the number of PEVs when imputing data as compared to the 2.1% increase using only complete data).The result for IQR increases in pollutants are shown in Additional file 1: Table S1.

## Discussion

In an area where air pollution levels most often comply with EU air quality guidelines, we observed associations between daily levels of air pollution the same day and the daily number of visits to a psychiatric emergency unit during the warm season (April to September). For a 10 μg/m^3^ increase in PM_10_, the number of PEVs increased by 3.6% (95% CI: 0.4–7.0%) in two-pollutant models. The three-pollutant model estimate was similar, but not statistically significant; 3.3%, (95% CI -0.2-6.9%). Our results add to existing evidence for air pollution to cause of mental distress or aggravate psychiatric disorders [12–14, 20, 25, 26].

The current study is one of the first studies of this kind and we used data that were readily available - an approach which has several limitations warranting discussion. First, due to a rather small study, statistical power was limited. Although the estimated increases in PEVs for the warm season (April to September) were very similar in the two-pollutant models (3.6%) compared to the multi-pollutant model (3.3%), the latter estimate was not statistically significant. Furthermore, patients with previous contact with the psychiatric emergency department there may seek health care there even for physical conditions. Therefore, diagnosis information is important to rule out that the association between air pollution and PEV’s are not driven by visits for physical symptoms. Data on age, sex, diagnoses and reason for visit would be desirable in order to better describe patient characteristics, and to conduct subgroup analyses. In order to increase statistical power and increase generalizability of the results, future studies should use data from other hospitals as well as demographic and diagnosis information in a multi-hospital study to disentangle possible associations between air pollution exposures and psychiatric disorders.

This study relied on exposure data from a single monitoring station as an exposure proxy for the residents of the whole Gothenburg area, which, although it is a commonly used approach in time-series studies, clearly is a limitation. There are many potential sources of exposure measurement errors in air pollution exposure time series studies, e.g. the correlation between ambient and indoor air pollution concentrations is only moderate [41], and so is the correlation between modelled outdoor residential exposure and measured exposure [42]. In time-series studies, differences between the average personal exposure and ambient measurements are the most likely source of substantial bias. [43]. Personal measurements of air pollution exposure is now feasible due to technical development, and increasingly used. Although they can certainly decrease measurement error and therefore help reduce attenuation of effect estimates, as measures of exposure or dose become more personal, the validity of study findings can decrease in ways that more proxy measures may avoid [44]. Measurement error could lead to a bias towards the null, if there was a true association between personal exposure and the outcome [44, 45]. Measurement error would however produce a false association only if monitor pollutant levels were overestimated on emergency room visit days (or underestimated on control days), which is unlikely. Personal behavior may affect exposure, for example if individuals with underlying mental disorders were less likely to leave their homes than individuals without such disorders, they would be less exposed. Furthermore, if their disorder was exacerbated on a day with high air pollution levels, the worsening could either cause them to not go out, thus avoiding exposure, or seek emergency care. Bias from personal behavior (not driven directly by the monitor level of the pollutant—or something confounding it) should however not account for the finding [44, 45].

The open-door policy of the clinic used in this study means that not all registered visits are emergencies, but we have little reason to believe that the proportion of non-emergency visits is associated with our exposure and it is estimated that less than 5% of all visits are due to such non-emergencies. Even if a visit is not a “true” emergency visit, but rather a visit for example for a prescription refill, it would associated with a worsening in some sense, following the same logic as studies on short-term effects of air pollution on dispensed medications [46].

A strength of time-series studies in general, in particular case-crossover studies, is that the risk of residual confounding from time invariant individual level factors such as life-style factors is negligible. That residual confounding—perhaps most likely by some other pollutant—could account for the association is possible, but it would have to be by something that varies within a calendar month. For a life-style factor to cause bias in a time-series study it would have to both vary temporally and be associated with the exposure of interest. Case-crossover designs adjust for individual level factors by design, and individual level confounding is thus very unlikely to explain the associations observed. However, in theory there could be residual confounding from some time-varying factor within the model strata (for example daily pollen counts) or meteorological variables that we, despite our efforts to do so in the statistical modelling, did not fully take into account. The estimates were very stable for any adjustments to seasonal and climate variables, which strengthens our results.

The association between PM_10_ and PEVs only seemed to be present during the warm season (April to September), and there was a tendency for NO_2_ to be associated with PEVs during the cold season (October to March), but not during the warm season. A possible explanation for the discrepancy could be that the composition of PM_10_ differs over the year and that NO_2_ (an indicator of traffic) “captures” the effect of PM_10_ during the cold season, where particles not believed to be associated with the outcome, such as suspended coarse particles from studded tires and sea salt are a large part of the PM_10_ mix. From that perspective, it would have been desirable to investigate PM_2.5_, since PM_2.5_ is usually a good marker for vehicle exhaust, but unfortunately a very large proportion of the PM_2.5_ observations were missing. It should be noted that the proportion of missing observations in the pollutants were much higher in the colder season that in the warm season, which may have diluted the estimates of associations. However, imputing missing data only marginally altered the estimates.

The association between PM_10_ and the number of PEVs was more evident during the warm season (April to September) where some hospital wards usually close for summer break, with more patients seeking care at the psychiatric emergency unit. If air pollution levels were higher in summer, such organizational changes would cause positive estimates as those in our study unless our study design fully adjusted for seasonal variation over time, a main reason for choosing the case-crossover design with control days selected within the same month. Therefore, such organizational changes most likely does not explain our results. Our study population was the entire Gothenburg municipality, and although any generalizations of our results to other study populations is uncertain, we see no major reason for our results not to be generalizable to populations with similar pollution-levels and socio-demographic compositions. Furthermore, reverse causation is not plausible given the study design.

The outcome in the present study was visits to the psychiatric emergency unit, which is a very broad marker of worsening in psychiatric disorders or of mental distress. A large part of the patients at the psychiatric emergency unit do not receive a psychiatric diagnosis per se but rather they suffer from distress related to life events such as bereavement or loss of job or house. Our data unfortunately does not allow us to distinguish between different causes of visits and hence our results do not allow us to speculate on which diagnoses or demographic subgroups that would be especially sensitive to air pollution exposure. There are very few previous studies on associations between acute air pollution exposure and worsening in psychiatric disorders or mental distress. Our results are however in line with the results from previous studies; Strahilevitz and colleagues [19] were the first to report associations between air pollution and psychiatric hospital visits in a study from a psychiatric hospital in St. Louis, USA. They reported correlations between mean daily levels of nitrogen dioxide (NO_2_) and carbon monoxide (CO) and emergency room visits by all patients [19]. In a study from 1983 Briere and colleagues observed a correlation between air pollution and schizophrenia and total visits to the psychiatric emergency room [17]. In 1984, Rotton and colleagues studied the relationship between calls to the police for assistance in handling psychiatric emergencies (also a broad marker of mental health) and levels of air pollution and observed associations which led them to conclude that psychiatric emergencies and treatment should be included in cost-benefit analyses of air pollution and its relationship to health [18]. More lately, in a study of six Canadian cities, Szyszkowicz and colleagues analyzed emergency department (ED) visits for depression and daily concentrations of CO, nitrogen dioxide (NO_2_), sulphur dioxide (SO_2_) and particulate matter (PM_10_) with a generalized linear mixed models technique. They observed statistically significant positive correlations between the number of visits for depression and several pollutants, for example a 15.5% (95% CI: 8.0–23.5) increase in ED visits per 0.8 ppm CO and 20.0% (95% CI: 13.3–27.2) per 20.1 ppb NO_2_, in the warmer period (April–September), which is similar to our results. For PM_10_, the largest increase however, 7.2% (95% CI: 3.0–11.6) per 19.4 μg/m3, was observed for the colder period (October–March) [26] whereas the estimates in our study were higher during the warmer season (April to September). In another study, Szyszkowicz and colleagues observed associations between CO, NO_2_, SO_2_ and PM_10_ concentrations and suicide attempts with the largest increase observed for males in the cold period for a 1-day lagged exposure to NO_2_, with an excess risk of 23.9% (95% CI: 7.8, 42.4). For the warm season, the associations were not statistically significant, in contrast to our results. Szyszkowicz and colleague have later reported associations between air pollution and ED visits for depression in another study, where they used a case-crossover study design, and where they observed that exposure to ozone was associated with increased risk of an ED visit for depression [25]. In a study from Korea, Cho and colleagues observed that SO_2_, PM_10_, NO_2_, and CO were positively associated with ED visits for depressive episode. The maximum risk was observed in the distributed lag 0–3 model for PM_10_ (Odds Ratio [OR], 1.120; 95% CI, 1.067–1.176) per standard deviation increase in pollutant for patients with either underlying cardiovascular disease, diabetes mellitus, asthma, or depressive disorder [27]. Furthermore, Bakian and colleagues observed associations between daily levels of air pollution and suicide completion, with an OR for an interquartile-range increase in nitrogen dioxide during cumulative lag 3 of 1.20, (95% CI: 1.04, 1.39) and fine particulate matter on lag day 2 by OR 1.05 (95% CI: 1.01, 1.10). In China, Chen and colleagues recently observed that a 10 μg/m3 increase in 2-day, moving-average concentration of inhalable particulate matter, sulfur dioxide (SO_2_), and carbon monoxide was associated with increments of 1.27% (95% CI: 0.28%, 2.26%), 6.88% (95% CI: 2.75%, 11.00%), and 0.16% (95% CI: 0.02%, 0.30%) in daily hospital admissions for mental disorders, (positive but not statistically significant associations were observed with PM_2.5_, O_3_ and NO_2_) [23]. The associations of air pollutants were generally stronger in the warm period (April to September), which is similar to our results. Thus there appears to be some discrepancy between studies with respect to which pollutant exhibits the strongest association, which outcome is used (although all outcomes to some extent reflect mental health) and whether associations have been observed all-year round or only in the cold season or warm season, as in the current study. Furthermore, there is some discrepancy regarding the lag of the association. Future studies should focus on resolving these discrepancies as well as investigating which psychiatric diagnosis, alternatively mental distress, are associated with bad air quality.

## Conclusions

In conclusion, our results imply that air pollution may be associated with a worsening in psychiatric disorders or mental distress in the warm season (April to September) in an area in Northern Europe where air pollution concentrations are generally well below the EU air quality guidelines. A large proportion of the world’s population is exposed to pollutant concentrations at similar or higher levels. Our results therefore imply that air pollution may be associated with larger human and societal costs than what is currently acknowledged. Given the scarcity of studies on acute air pollution and associations with worsening in psychiatric disorders or mental distress, and the borderline statistical estimates of our study, our results should however be corroborated by others.

## Additional file

Additional file 1: Figure S1. QQ-Plots of the residuals for single pollutant models. **Figure S2.** QQ-Plots of the residuals for multiple pollutant models. **Figure S3.** Correlations between air pollutants. **Table S1.** Percent change in the number of Psychiatric Emergency Visits (PEVs) with their 95% Confidence Intervals for a lag 0 IQR increase of the air pollutant for Single and Multi-pollutant models during the whole year, during the warmer season (April to September) and during the colder season (October to March). (DOCX 435 kb)

## Abbreviations

CI: Confidence Interval
CO: Carbon monoxide
ED: Emergency department
GDP: Gross Domestic Product
HR: Hazard Ratio
NO_2_: Nitrogen dioxide
O_3_: Ozone
OECD: Organisation for Economic Co-operation and Development
OR: Odds Ratio
PEV: Psychiatric Emergency Department Visit
PM_10_: Particulate matter with an aerodynamic diameter ≤ 10 μg^−3^
PM_2.5_: Particulate matter with an aerodynamic diameter ≤ 2.5 μg^−3^
QAIC: Akaike Information Criteria for Quasi Poisson
SO_2_: Sulphuric dioxide
