# Supplementary material for: The association between daily concentrations of air pollution and visits to a psychiatric emergency unit: a case-crossover study
Source: Environ Health. 2018 Jan 10;17:4. doi: 10.1186/s12940-017-0348-8 (PMC5763570; doi:10.1186/s12940-017-0348-8)
Supplement: Additional file 1: Figure S1. — QQ-Plots of the residuals for single pollutant models. Figure S2. QQ-Plots of the residuals for multiple pollutant models. Figure S3. Correlations between air pollutants. Table S1. Percent change in the number of Psychiatric Emergency Visits (PEVs) with their 95% Confidence Intervals for a lag 0 IQR increase of the air pollutant for Single and Multi-pollutant models during the whole year, during the warmer season (April to September) and during the colder season (October to March). (DOCX 435 kb) [file 12940_2017_348_MOESM1_ESM.docx]

Figure S1. QQ-Plots of the residuals for single pollutant models.


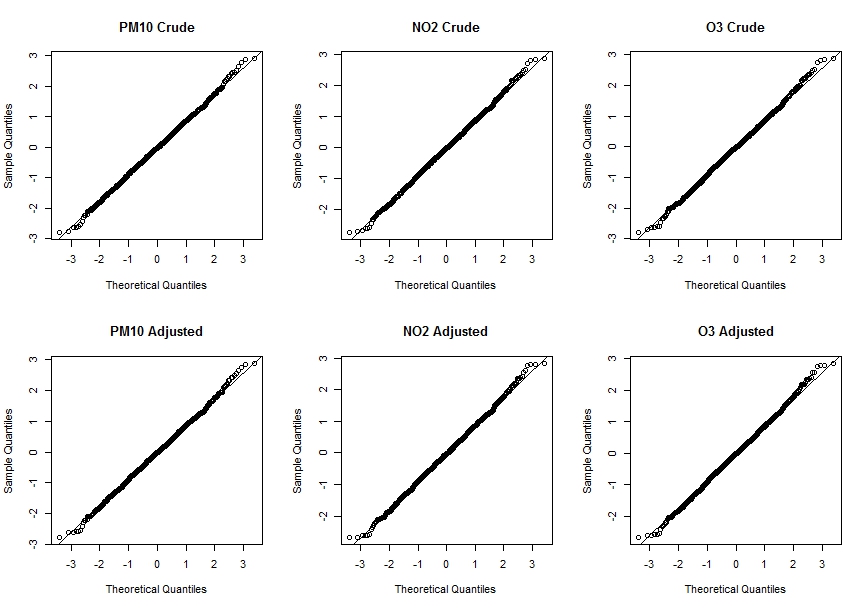


Figure S2. QQ-Plots of the residuals for multiple pollutant models.


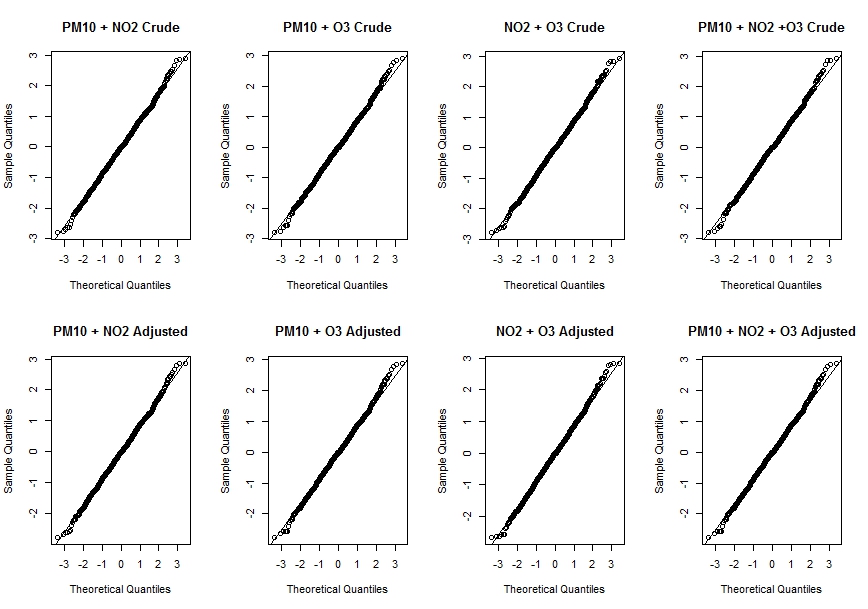


Figure S3. Correlations between air pollutants.


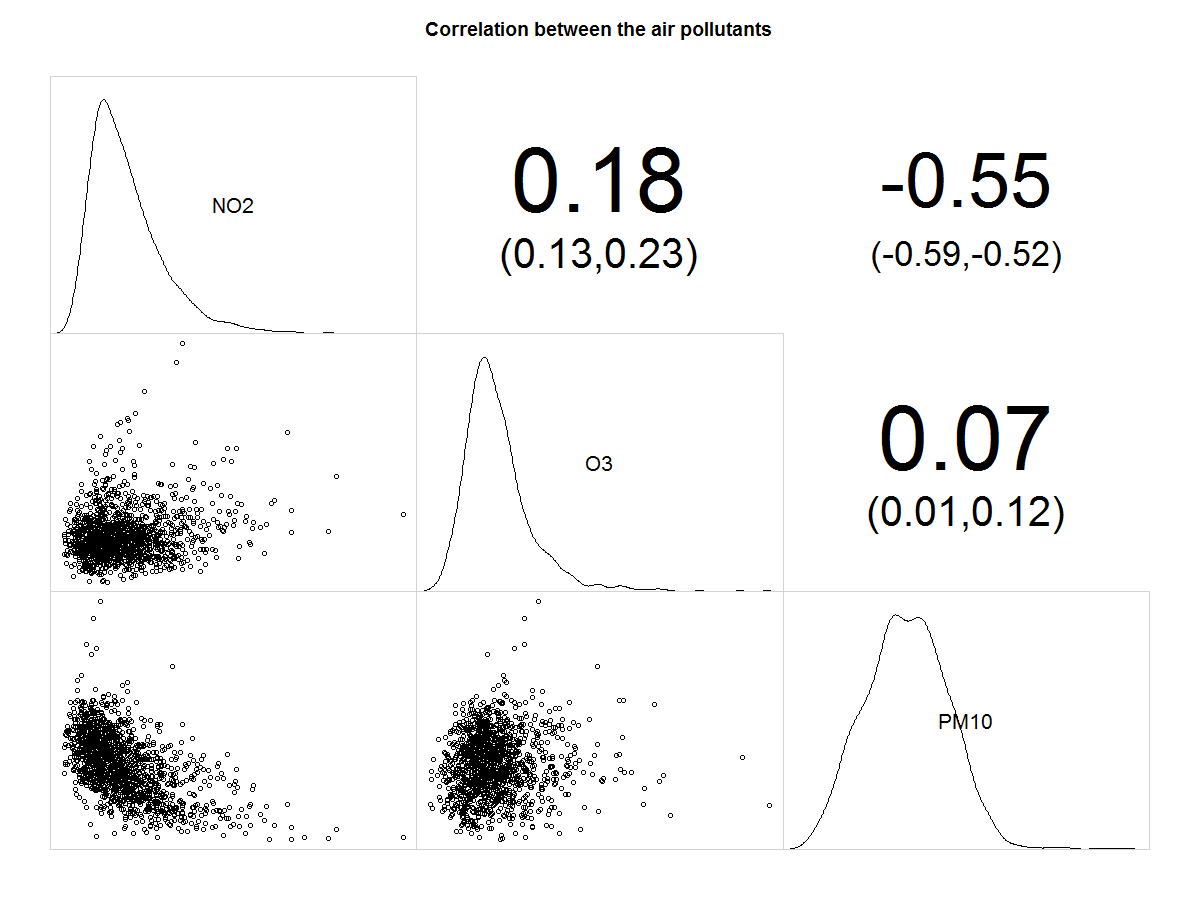


| Table S1. Percent change in the number of Psychiatric Emergency Visits (PEVs) with their 95% Confidence Intervals for a lag 0 IQR increase of the air pollutant for Single and Multi-pollutant models during the whole year, during the warmer season (April to September) and during the colder season (October to March). | | | | |  |
| --- | --- | --- | --- | --- | --- |
|  |  | PM_10_ | NO_2_ | O_3_ |  |
|  | Model | Change PEVs (%) (95% CI) | Change PEVs (%) (95% CI) | Change PEVs (%) (95% CI) |  |
| Single Pollutant | Crude^1^ | 1.4 (0.1-2.7) | 0.6 (-1.1-1.8) | 0.5 (-1.6-2.6) |  |
| Single Pollutant | Adjusted^2^ | 1.5 (0.2-2.9) | 0.4 (-1.0-2.0) | 0.4 (-1.8-2.5) |  |
|  |  |  |  |  |  |
| Two-pollutants |  |  |  |  |  |
| PM_10_ + NO_2_ | Crude | 1.3 (0.0-2.7) | 0.6 (-1.0-2.2) |  |  |
| PM_10_ + NO_2_ | Adjusted | 1.5 (0.1-2.8) | 0.6 (-1.0-2.2) |  |  |
| PM_10_ + O_3_ | Crude | 1.1 (-0.3-2.6) |  | 0.9 (-1.3-3.1) |  |
| PM_10_ + O_3_ | Adjusted | 1.3 (-0.2-2.7) |  | 0.8 (-1.4-3.1) |  |
| NO_2_ + O_3_ | Crude |  | 1.0 (-0.9-3.0) | 1.2 (-1.5-3.9) |  |
| NO_2_ + O_3_ | Adjusted |  | 1.0 (-0.9-3.0) | 1.1 (-1.6-3.8) |  |
| Three-pollutants |  |  |  |  |  |
| PM_10_ + NO_2_ + O_3_ | Crude | 1.0 (-0.5-2.5) | 1.6 (-0.6-3.7) | 2.1 (-0.8-5.0) |  |
| PM_10_ + NO_2_ + O_3_ | Adjusted | 1.1 (-0.4-2.6) | 1.5 (-0.7-3.7) | 1.9 (-1.0-4.9) |  |
|  |  |  |  |  |  |
| *Warmer season (April to September)* | | | | |  |
| Single Pollutant | Crude^1^ | 2.3 (0.2-4.4) | -0.3 (-2.7-2.2) | 1.9 (-0.9-4.8) |  |
| Single Pollutant | Adjusted^2^ | 2.4 (0.3-4.5) | 0.0 (-2.4-2.5) | 1.6 (-1.3-4.5) |  |
|  |  |  |  |  |  |
| Two-pollutants |  |  |  |  |  |
| PM_10_ + NO_2_ | Crude | 2.4 (0.2-4.6) | -0.7 (-3.1-1.8) |  |  |
| PM_10_ + NO_2_ | Adjusted | 2.4 (0.3-4.6) | -0.5 (-2.9-2.1) |  |  |
| PM_10_ + O_3_ | Crude | 2.0 (-0.1-4.2) |  | 1.3 (-1.6-4.1) |  |
| PM_10_ + O_3_ | Adjusted | 2.1 (-0.1-4.3) |  | 0.9 (-1.9-3.9) |  |
| NO_2_ + O_3_ | Crude |  | 0.8 (-2.1-3.8) | 2.4 (-0.9-5.9) |  |
| NO_2_ + O_3_ | Adjusted |  | 0.9 (-2.0-3.9) | 2.2 (-1.2-5.6) |  |
| Three-pollutants |  |  |  |  |  |
| PM_10_ + NO_2_ + O_3_ | Crude | 2.1 (-0.2-4.5) | -0.2 (-3.2-3.0) | 1.2 (-2.3-4.8) |  |
| PM_10_ + NO_2_ + O_3_ | Adjusted | 2.2 (-0.1-4.6) | -0.1 (-3.2-3.1) | 0.9 (-2.6-4.6) |  |
|  |  |  |  |  |  |
| *Colder season (October to March)* | | | | |  |
| Single Pollutant | Crude^1^ | 0.9(-1.8-2.6) | 0.7 (-1.1-2.5) | -1.2(-4.3-2.0) |  |
| Single Pollutant | Adjusted^2^ | 1.1 (-0.6-2.8) | 0.6 (-1.3-2.5) | -1.0 (-4.2-2.4) |  |
|  |  |  |  |  |  |
| Two-pollutants |  |  |  |  |  |
| PM_10_ + NO_2_ | Crude | 0.8 (-1.0-2.5) | 1.3 (-0.8-3.5) |  |  |
| PM_10_ + NO_2_ | Adjusted | 1.0 (-0.8-2.8) | 1.1 (-1.1-3.3) |  |  |
| PM_10_ + O_3_ | Crude | 0.5 (-1.5-2.4) |  | 0.1 (-3.5-3.7) |  |
| PM_10_ + O_3_ | Adjusted | 0.7 (-1.3-2.7) |  | 0.6 (-3.1-4.4) |  |
| NO_2_ + O_3_ | Crude |  | 0.7 (-2.0-3.4) | -0.8 (-5.1-3.6) |  |
| NO_2_ + O_3_ | Adjusted |  | 0.6 (-2.2-3.5) | -0.7 (-5.1-3.9) |  |
| Three-pollutants |  |  |  |  |  |
| PM_10_ + NO_2_ + O_3_ | Crude | 0.3 (-1.7-2.3) | 2.6 (-0.6-5.8) | 2.6 (-2.6-8.0) |  |
| PM_10_ + NO_2_ + O_3_ | Adjusted | 0.5 (-1.5-2.6) | 2.5 (-0.8-5.9) | 3.0 (-2.3-8.6) |  |
|  |  |  |  |  |  |
| ^1^Model with only air pollutants  ^2^Adjusted = Model adjusting for daily mean temperature (continuous) and public Swedish holiday (Yes/No) | | | | | |
